# Lithium-ion battery waste as a robust oxygen evolution reaction electrocatalyst for seawater splitting

**DOI:** 10.1038/s41598-025-34856-w

**Published:** 2026-01-12

**Authors:** Magdalena Warczak, Katarzyna Belka, Weronika Urbańska, Monika Michalska, Njemuwa Nwaji, Magdalena Osial

**Affiliations:** 1 Faculty of Chemical Technology and Engineering, Bydgoszcz University of Science and Technology, Seminaryjna 3 Street, Bydgoszcz, 85-326 Poland; 2 Faculty of Environmental Engineering, Wrocław University of Science and Technology, Wybrzeże Wyspiańskiego 27 Street, Wrocław, 50-370 Poland; 3 Faculty of Materials Science and Technology, Department of Chemistry and Physico-Chemical Processes, VSB-Technical University of Ostrava, 17. listopadu 2172/15, Ostrava-Poruba, 708 00 Czech Republic; 4 Institute of Fundamental Technological Research, Department of the Theory of Continuous Media and Nanostructures, Polish Academy of Sciences, Pawińskiego 5B Street, Warsaw, 02-106 Poland

**Keywords:** Lithium-ion battery, Waste, Electrocatalysis, Oxygen evolution, Seawater splitting, Electrochemistry, Energy, Electrocatalysis

## Abstract

Electrocatalytic seawater splitting seems to be the most promising and urgent demand strategy for clean hydrogen energy production. Utilizing low-cost electrocatalysts is pivotal in the hydrogen economy, as seawater splitting can be made highly efficient and more economical. To meet these expectations, we proposed a novel utilization for the black carbon mass left over from hydrometallurgical metal recovery as an efficient and stable electrocatalyst for oxygen evolution reaction (OER) performed in alkaline media. The SEM-EDS, XPS, XRD, XRF, and Raman analyses revealed that the composition and structure of the post-leached battery powders depend on the hydrometallurgical waste recycling conditions, which in turn affect their OER electrocatalytic activity. In particular, the material leached with sulfuric acid (BAT 1) retained a higher content of cobalt-based compounds (mainly LiCoO_2_ and Co_3_O_4_) embedded within a porous carbon matrix and, resulting in the best catalytic performance among all samples. The enhanced performance of BAT 1 is attributed to the synergy of its cobalt-based phases and the well-developed porous carbon structure, which collectively result in a high electrochemically active surface area. The electrochemical tests proved that Li-ion battery waste has remarkable OER catalytic performance with an overpotential of 226 mV and 225 mV, reaching 10 mA cm^− 2^ in water splitting and in seawater splitting, respectively, which is only 14 mV and 95 mV higher than for benchmark RuO_2_ in water splitting and seawater splitting, respectively.

**Supplementary Information:**

The online version contains supplementary material available at 10.1038/s41598-025-34856-w.

## Introduction

 Achieving net-zero emissions is increasingly being realized through the widespread adoption of electric vehicles (EVs). However, effective end-of-life (EOL) management of lithium-ion batteries (LIBs) remains a significant challenge^1–3^. It is expected that the end of life of millions of LIBs will soon be in sight, with projections for the EV market to be 530 million vehicles by 2040^4^. Besides, this challenging issue is exacerbated by the rising incidence of excessive use of portable electronics. The cathode component of these LIBs contains useful elements, including critical raw materials such as Ni, Mn, Co, and Li, which can be recovered via recycling^5^. The lack of effective recycling poses a serious environmental and health risk, with less than 0.2 million tonnes of LIB waste recycled globally in 2019, most of which comes from portable electronics rather than electric vehicles^6,7^. Inappropriate disposal, for example, in landfills, may cause toxic metals such as cobalt to leach into the environment and contaminate soil and water, endangering human health. To maximize the recovery and reuse of raw materials, the development of a sustainable battery value chain that includes efficient EOL recycling is urgently needed^8^. Although various efforts have been made to recover Li, Ni, Mn, and Co (Li-NMC) from spent LIB cathodes, reusing the recycled form in new LIBs is technologically challenging^7,9^. Due to its environmentally friendly, efficient, and sustainable nature, electrochemical water splitting, involving hydrogen evolution reaction (HER) at the cathode and oxygen evolution reaction (OER) at the anode, has received a lot of attention^10,11^. However, the significant use of fresh water in the large-scale electrolysis of water is a cause for concern for water resources. Harnessing green hydrogen from seawater electrolysis is a key strategy for achieving dual-carbon goals, as seawater accounts for 96.5% of the world’s water^12,13^. Direct seawater splitting streamlines the process by direct hydrogen production from seawater, unlike indirect seawater electrolysis, which requires desalination. However, the significant challenge at the anode in direct seawater electrolysis is oxidation of the high concentration of chlorine ions (Cl^−^) to hypochlorite (ClO^−^)^14,15^,, which poses a threat to electrode durability due to the corrosive nature of these products. The energy efficiency of the seawater splitting process would be significantly reduced by the electrode corrosion and poisoning caused by the byproducts of the aforementioned anodic reactions. From a thermodynamic point of view, OER is more favourable over the entire pH range, especially in alkaline media, where the difference of standard electrode potential between OER and hypochlorite formation remains a constant value of 480 mV. However, the kinetics of chlorine evolution reaction/hypochlorite evolution reaction (ClER/HCER) makes it more facile than OER. Therefore, the approach of designing the electrocatalysts for alkaline seawater splitting should be focused on materials that exhibit OER overpotential lower than 480 mV, leading to HCER suppression^16,17^. In addition, seawater is a complex medium that contains a variety of inorganic salts, bacteria, microplastics, and dissolved gasses that can poison electrodes and hamper their long-term stability and durability, as well as that of electrocatalysts, membranes, and other materials in the seawater electrolyser. To overcome these issues, a number of strategies are utilized, including the electrocatalysts’ structure and composition design, their surface modification engineering, as well as the local environment customization, ensuring high catalyst performance, stability, and selectivity^16,18^.

To date, noble metal-based compounds such as ruthenium and iridium have been thought to be the most efficient and selective OER electrocatalysts in seawater splitting^19^. However, their scarcity and elevated cost restrict their widespread technological use. Therefore, non-noble metal-based materials such as transition metal oxides, carbides, phosphides, sulfides, selenides, and chalcogenides are widely exploited as OER electrocatalysts^20–28^. And, the combination of transition metal compounds and carbon-based materials leads to improved dispersion of active sites and enhanced catalytic activity of such composites^29^. Therefore, the exploration of such low-cost, stable, and highly active carbon-based transition metal compounds seems to be a reasonable approach for designing not only OER electrocatalysts in seawater splitting but also in general in energy conversion systems^30^.

Following this strategy, recently, we demonstrated that lithium-ion battery waste has excellent electrocatalytic activity for ORR to H_2_O_2_ generation^31^. Furthermore, we revealed the impact of the structure and composition of the battery waste on its ORR catalytic capabilities^32^. And, in this work, we show the electrocatalytic performance of battery waste toward OER in the water splitting process. Furthermore, these studies provide the first new evidence that the carbon black mass left over from the leaching process of the battery waste could be a potential OER electrocatalyst in seawater splitting. Moreover, in contrast to previous studies^33,34^ the utilization of the raw battery waste material without any additional pretreatments is a significant improvement of the Li-ion battery waste recycling and circular economy and opens new paths for its applications in energy conversion systems.

## Experimental section

### Chemicals

Aquivion solution (25% in water), RuO_2_ powder, LiCoO_2_ powder, sodium chloride (NaCl), and glutaric acid (C_5_H_8_O_4_) (analytical grade), (Merck KGaA) were purchased from Sigma Aldrich. KOH (analytical grade) was supplied by POCH, H_2_O_2_ (30% analytical grade), and H_2_SO_4_ (96% analytical grade) were received from STANLAB (Lublin, Poland). Deionized water purified with HYDROLAB (Gliwice, Poland) with ion columns was used to prepare solutions for the electrochemical studies.

### Acid-leaching method for metals’ recovery from spent Li-ion batteries

First, spent LiBs collected from laptops of various manufacturers (including Toshiba, Samsung, and Asus) were mechanically dismantled, and the anodes and the cathodes were separated from other fractions before being crushed and ground into powder. Then, the powders were washed with distilled water, and dried in an oven overnight at 90 °C. The battery waste powders were then treated under different conditions, where the samples were named BAT 1, BAT 2, and BAT 3 (see Table 1). To prepare BAT 1 sample, the powder was treated with 1.5 M sulfuric acid (H_2_SO_4_) with a mass ratio of 1:10 (solid residue: liquid) for 120 min with the mechanical stirring of about 500 rpm. In the case of material BAT 2: the solid residue was treated with 5 M formic acid (CH_2_O_2_) at 55 °C for 3 min with magnetic stirring at 500 rpm and then 5 g of glutaric acid (C_5_H_8_O_4_) and 3 mL of 30%_aq_ hydrogen peroxide (H_2_O_2_) were added with continuous stirring for an additional 120 min. Then, the residue was filtered to separate the solid residue from the leaching bath. The post-leaching residue (the carbon black mass) was then rinsed with deionized water until neutralization, dried at 50 °C overnight, mechanically ground, and then used for electrochemical studies. In the case of material BAT 3: instead of 5 M formic acid (CH₂O₂), lactic acid (C_3_H_6_O_3_) was used with the same procedure. Figure S1 displays the rate of metal recovery from battery waste as a result of leaching process operating under various conditions, leading to producing black carbon battery waste masses: BAT 1–3 (see Table 1).

**Table 1:** Leaching baths for battery waste materials BAT 1–3.

BAT 1	BAT 2	BAT 3
1.5 M H_2_SO_4_ + 0.9% v/v H_2_O_2_	5.0 M CH_2_O_2_ + 0.38 M C_5_H_8_O_4_ + 0.9% v/v H_2_O_2_	5.0 M C_3_H_6_O_3_ + 0.38 M C_5_H_8_O_4_ + 0.9% v/v H_2_O_2_

### Characterization

The morphology analysis was performed using ZEISS Crossbeam 350 scanning electron microscope (SEM) equipped with X-ray Electron Dispersive Spectroscopy (EDS), Zeiss, Germany.

The SPECS PHOIBOS 100 hemispherical analyser with a 5-channel detector and a SPECS XR50/FOCUS 500 monochromatic X-ray source equipped with an Al and Ag dual anode was used to analyse the samples’ surface composition and the chemical state of the elements. The Al anode at E_pass_ 40 eV and 10 eV was used for survey and high-resolution spectra, respectively. The spectra were collected in a normal direction, and a sample charge was compensated by the SPECS FG22 flood gun during the measurements. The analyser was set to work in Fixed Analyzer Transmission mode and Medium Area (Magnification M = 5) settings with an entrance slit of 7 × 20 mm^2^ and Iris diameter of 35 mm, thus the measured area is about 1.4 × 4 mm^2^. The pressure was kept under 7 × 10^− 7^ Pa during the measurements. The acquired data were processed in CasaXPS software with a Shirley background profile and built-in RSF was used for the calculation of the elemental composition.

X-ray fluorescence spectroscopy (XRF) was conducted on the wave-dispersive XRF spectrometer Rigaku Primus IV using standardless SQX analysis based on the fundamental parameters method. This spectrometer enables the measurement of element concentration across the range from F to U, with a concentration range of 1 ppm to 100%.

The X-ray diffractometry (XRD) was performed on a Panalytical X’Pert Pro MPD (Multipurpose Diffractometer). Data collection was performed over a range from 10 to 90° with a scanning rate of 1.5° (2θ)/min with CuKα radiation (45 kV, 40 mA, λ = 1.5406 nm). The crystal phases were identified by referencing diffraction patterns in a licensed library from the International Centre for Diffraction Data (ICDD).

The Raman spectra were acquired on a DXR Raman microscope (Thermo Scientific) with a 32-two-second scan, laser 532 nm (3 mW) under a 10 × objective of an Olympus microscope.

The content of heavy metal ions in the solution after the leaching to determine the recovery rate was investigated using an inductively coupled plasma mass spectrometer (ICP-MS) NexION 5000 Perkin Elmer (USA).

### Electrocatalytic activity measurements

Linear sweep voltammetry (LSV), cyclic voltammetry (CV), chronopotentiometry (CP), and electrochemical impedance spectroscopy (EIS) were conducted with an Ivium potentiostat (Ivium Technologies, Netherlands) in a three-electrode cell. The static glassy carbon (GC) disc electrode (0.0314 cm^2^, Mineral, Poland) was employed as a working electrode, while the Hg/HgO electrode (Mineral, Poland) and Pt wire (Mineral, Poland) served as a reference electrode and a counter electrode, respectively. All potentials were recalculated vs. the RHE electrode, referring to the Nernst equation where E^0^_Hg/HgO_= 0.098 V^36^. The GC electrodes were modified with a suspension of 2.5 mg battery waste powder in 10 µL of 5% Aquivion solution (25% Aquivion solution in water mixed with isopropanol resulting in 5% ionomer solution). Electrochemical experiments were conducted in 0.1 M KOH solution (pH 13.16) or 1 M KOH + 1 M NaCl (1:1 vol., pH 12.56) under ambient conditions. The double layer capacitance C_dl_ was determined using CV recorded at different scan rates in the non-faradaic potential region (ΔE). C_dl_ was calculated from the equation: Δj = (j_a_-j_c_)/2ν, where j_a_ and j_c_ are the anodic and cathodic current densities at ΔE and ν is the scan rate in mV s^− 1^^37^. Electrochemical impedance spectra were recorded in the frequency range of 10 kHz–0.1 Hz with an alternating AC voltage of 10 mV amplitude. The EIS results were analyzed by ZView ver. 4.1b software (Scribner Associates, Inc.).

## Results and discussion

### The morphology and composition analysis

**Fig. 1:**
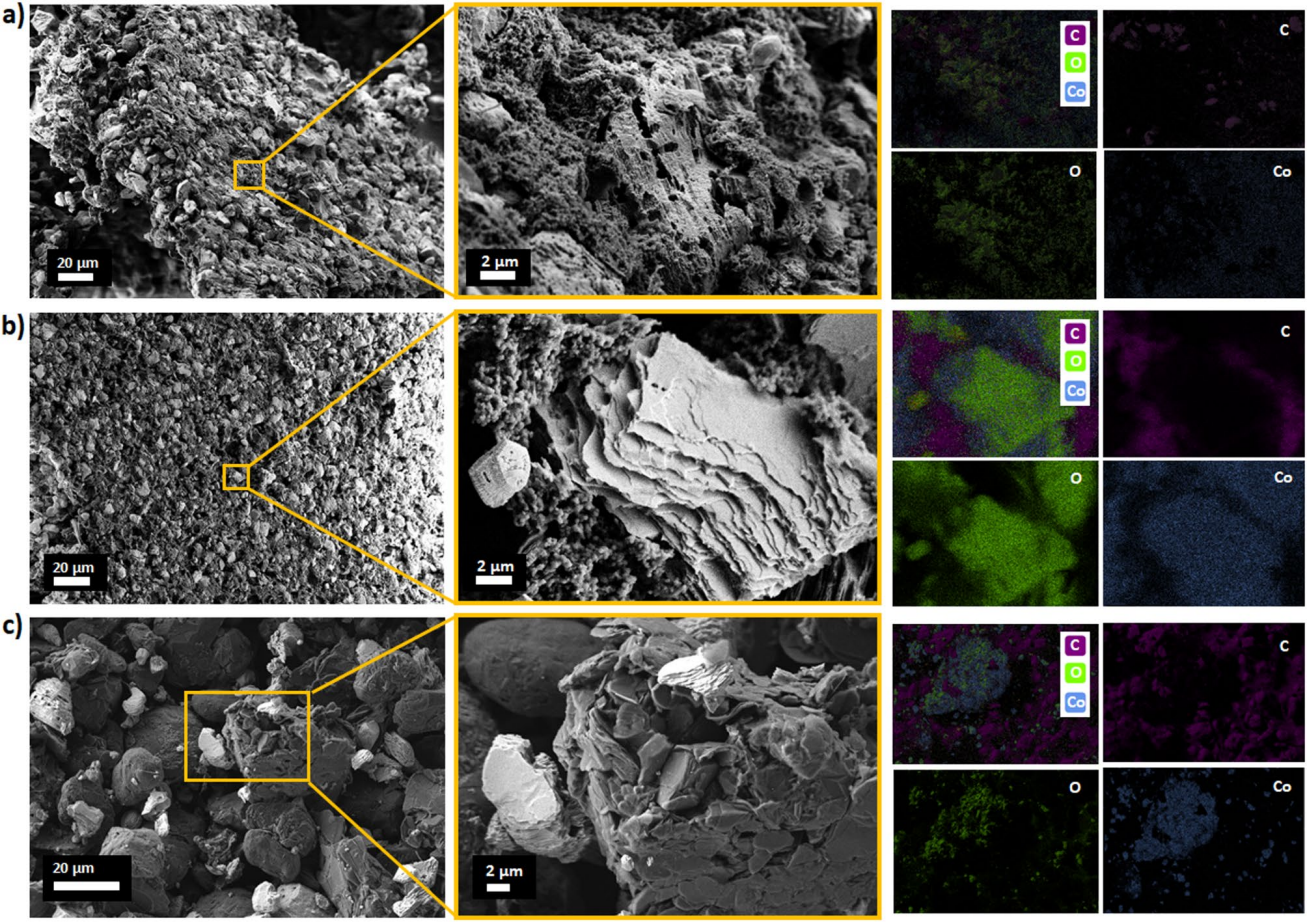
SEM images and EDS maps of battery waste materials: (**a**) BAT 1; (**b**) BAT 2, and (**c**) BAT 3.

Morphology analysis using scanning electron microscopy revealed differences in the battery waste powders leached under various experimental conditions (Table 1). As can be seen in Fig. 1a, the BAT 1 sample has a highly porous surface, differing from the other samples, where layered particles are stacked on top of each other, also containing fine particles with a non-uniform shape on the surface. The fine structures can be attributed to the presence of cobalt oxide-based structures, while the surrounding larger structures are derived from carbon. The following BAT 2 sample (Fig. 1b) has a heterogeneous structure in which flat, flake-like, multilayered clusters are visible along with granular nanostructures surrounding larger flakes on the micron-scale carbon grains. Elemental mapping using EDS indicates that the granules can be attributed to a carbon-based matrix in which larger structures containing cobalt oxide-based structures can be distinguished. The presence of granules may be related to the different leaching conditions. EDS analysis for BAT 1 also revealed the presence of sulfur as a result of leaching the battery waste in sulphuric acid (Figure S2).

The morphology of the BAT 3 sample is granular, where one can see clumped, flat structures covered with numerous, finer objects with irregular shapes and porous surfaces; see Fig. 1c. The EDS map shows the aggregates of the cobalt-based compounds randomly dispersed onto the carbon-based matrix. Among these three samples, BAT 1 shows the most porous and complex structure that can be related to the promising catalytic properties.

**Fig. 2:**
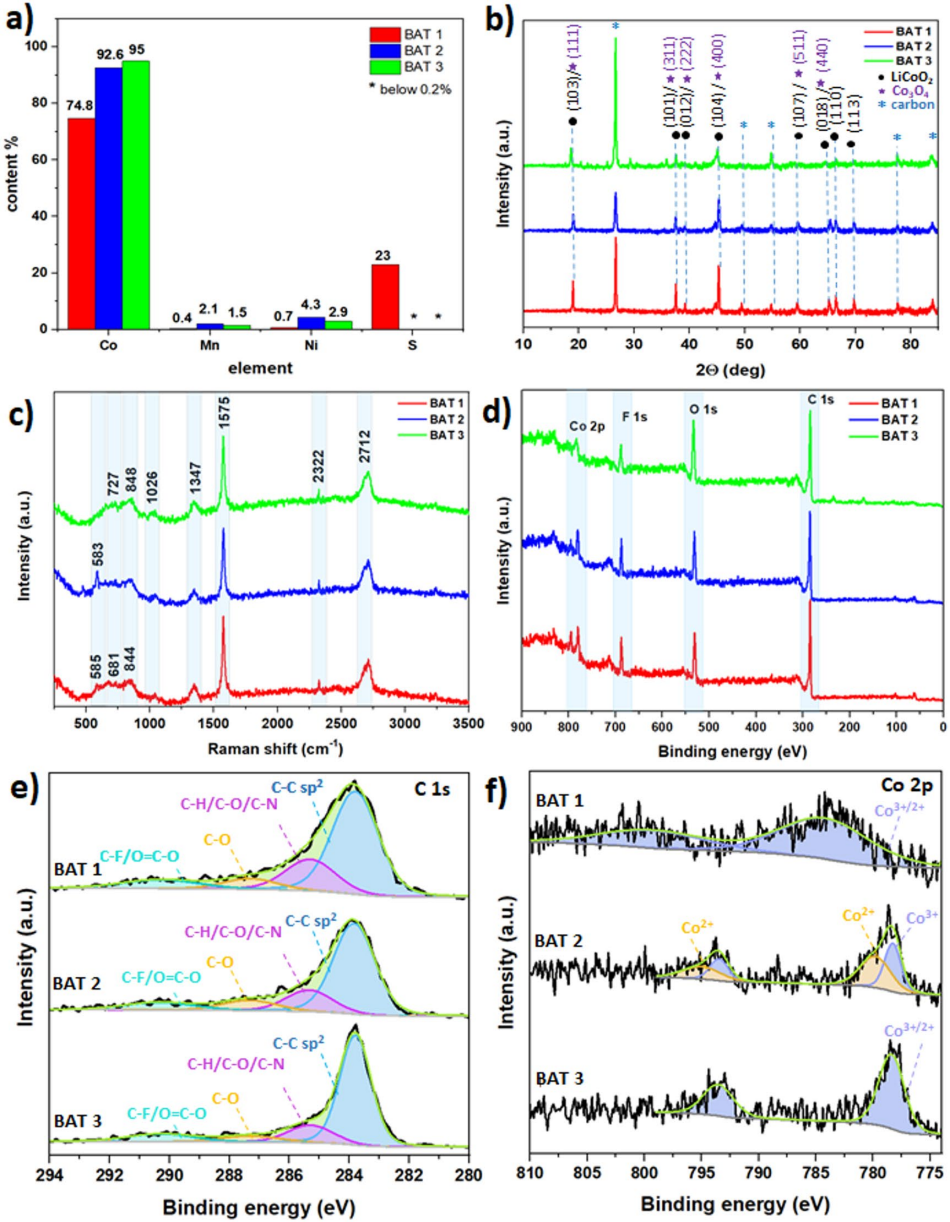
(**a**) XRF results, (**b**) XRD pattern, (**c**) Raman spectra, (**d**) XPS survey spectra, (**e**) C 1 s spectra, and (**f**) Co 2p.

Upon completion of the SEM-EDS analysis, the X-ray fluorescence (XRF) technique was employed to ascertain the metals’ contents (in mass percentages) in the post-leached battery waste powders BAT 1–3. The XRF study reveals that cobalt is the main metal in all tested materials (Fig. 2a). Its mass percentage ranges from 74.8% for BAT 1 up to 92.6% and 95% for BAT 2 and BAT 3, respectively. While nickel, manganese, and copper were also found, their mass percentages in the tested samples were below a few percent (Fig. 2a). As BAT 1 was leached with sulfuric acid, a signal from possible sulfur compounds, e.g., CoSO_4_, is evident in this sample. Given that the XRF analysis is only an elemental technique, the samples were subjected to the following further examinations.

The crystallinity of BAT 1–3 samples was studied by X-ray diffraction (XRD). The XRD patterns in Fig. 2b show only slight changes in peak intensity, indicating that leaching under different experimental conditions slightly affects the battery waste powder. It can be seen that the peaks located at 2θ = 18.54, 35.9, 37.6, 45.3, 59.5, 65.2̊ can be ascribed to the Co-based materials, in particular LiCoO_2_^38,39^ assigned to the (103), (101), (012), (104), (107), (018), (110) (JC-PDS 00–075-0532 for LiCoO_2_) and/or cobalt oxides such as Co_3_O_4_ ascribed to the (111), (311), (222), (400), (511), (440) (JC-PDS 00–042-1467 for Co_3_O_4_)^38,39^, respectively. The peaks located at 2θ = 26.6, 49.6, 54.7, 77.6, and 83.9̊ can be ascribed to the graphitic carbon, where the lowest intensity is recorded for BAT 2 among the BAT 1*–*3 samples. This effect relates to the leaching conditions, where the application of the mild organic acids instead of strong inorganic acids leach metals such as cobalt with the various yield.

The peaks located at 2θ = 26.6, 49.6, 54.7, 77.6, and 83.9̊ can be ascribed to the graphitic carbon, where the lowest intensity is recorded for BAT 2 among the BAT 1*–*3 samples. This effect relates to the leaching conditions, where the application of the mild organic acids instead of strong inorganic acids leach metals such as cobalt with the various yield.

The Raman spectroscopy analysis clearly shows the differences in the stoichiometry between the peaks for the particular ingredients, including graphitic carbon, cobalt oxides, and pristine LiCoO₂ that was not fully leached. The highest peaks intensity relating to the carbon is recorded for the sample BAT 3. The presence of pristine LiCoO₂ is observed in BAT 2 and BAT 3, where a peak at 583 cm⁻¹, attributed to the E_g_ mode of LiCoO₂, is detected (see Fig. 2c)^39^.

These findings are in accordance with the results of the X-ray diffraction (XRD) studies. However, due to the high noise-to-signal ratio in the Raman spectra, the expected peak at approximately 483 cm⁻¹, ascribed to the A_1g_ mode of LiCoO₂ in the literature, is barely discernible.

The signal observed in the range of 590–800 cm^− 1^, which is associated with M-O vibrations, may be attributed to delithiated LiCoO_2_. [40] and/or Co_3_O_4_ and/or Co_3_O_4_^40,41^. It can thus be inferred that the samples contain a mixture of delithiacted and pristine LiCoO₂ as well as cobalt oxide. The peaks with the highest intensities, at approximately 1347, 1575, 2322, and 2712 cm^− 1^, are attributed to the D, G, 2D, and D + G bands of graphitic carbon. The observed peaks at 848 and 1026 cm^− 1^ are likely attributed to electrolyte residues that may have been trapped within the pore structures of the materials. These findings are consistent with those reported elsewhere^42,43^. Furthermore, the presence of other metals, such as Mn and Ni, in BAT 1*–*3 may also contribute to the observed signals, given the potential for these elements to form oxides. It was observed that the surface of the BAT 1–3 samples differed in composition from the bulk. In order to determine the leaching effect on the chemical composition of the battery waste powders, X-ray photoelectron spectroscopy (XPS) was employed. XPS survey spectra performed for BAT 1*–*3 materials (Fig. 2d) confirmed the presence of carbon, oxygen, cobalt, and fluorine in all tested samples as well as additional sulfur for BAT 1, which was leached using sulfuric acid.

The high-resolution spectra reveal distinct peaks corresponding to C = C, C-C, C-O, and O = C-O bands in the materials, as shown in the C 1 s spectrum (Fig. 2e). While the C 1 s spectra are generally similar across the samples, a slight variation is observed in BAT 2, where the peak for C = C is lower compared to BAT 1 and BAT 3. The C 1 s peaks within the binding energy range of 284 eV to 291 eV are characteristic of graphite, the primary constituent of the anodes in Li-ion batteries^44^. The peak at 284 eV, which exhibits the highest intensity, is attributed to C-C bonds in the graphite sheets, which are sp^2^ hybridized^45^. Peaks between 285 eV and 287 eV are indicative of carbon with sp³ hybridization, bonded to heteroatoms such as C- H, C-O, or C-N.^47^ Additionally, the peak at 291 eV suggests the presence of carbonates and/or C-F bonds, which may arise from trace amounts of the LiPF_6_electrolyte^48,49^,, or potentially from O = C-O bonds formed by the creation of –COOH groups on the carbon surface during acid leaching^50–52^. Fig. 2f presents the valence spectrum for a Co-based compound, where spin-orbit coupling results in two distinct Co 2p_3/2_ and Co 2p_1/2_ peaks for BAT 1 and BAT 3. However, the peaks for BAT 3 are notably broader and shifted toward higher binding energies. The deconvolution spectra reveal two prominent peaks, which are attributed to compounds such as CoO, Co₃O₄, Co(OH)₂, and Co₂O₃ in the BAT 1 and BAT 2 samples^52,53^. For BAT 3, these peaks are shifted to binding energies of approximately 785 eV and 800 eV, corresponding to the Co 2p_3/2_ and Co 2p_1/2_ orbitals, respectively. This shift suggests the presence of Co²⁺ and Co³⁺ ions, likely originating from CoSO₄, CoO, and Co₃O₄ compounds^52^. The deconvolution curves for BAT 2 further indicate an additional pair of peaks, which may imply the coexistence of both Co³⁺ and Co²⁺ in the sample^54^. Additionally, the formation of CoF₂ can also be inferred^55^.

The supplementary material presents the high-resolution spectra for O 1 s, F 1 s, and S 2p spectra, as shown in Figure S3. The O 1 s XPS spectra reveal peaks at 531–534 eV that can be attributed to the C = O bonding and to the graphite C-O surface groups resulting from surface modification by the leaching process^47^. The appearance of F 1 s peaks at approximately 688–689 eV indicates the presence of C-F bonds and LiFP_6_^48^ in the BAT 1*–*3 samples. Additionally, the S 2p peaks observed in BAT 1 between 169 and 173 eV are likely associated with S-F bonds or CoSO_4_^53^. As BAT 1 is the only sample subjected to leaching with sulfuric acid, the sulfur-based peak is observed exclusively in this sample.

### Electrochemical characterization

#### Water splitting

**Fig. 3:**
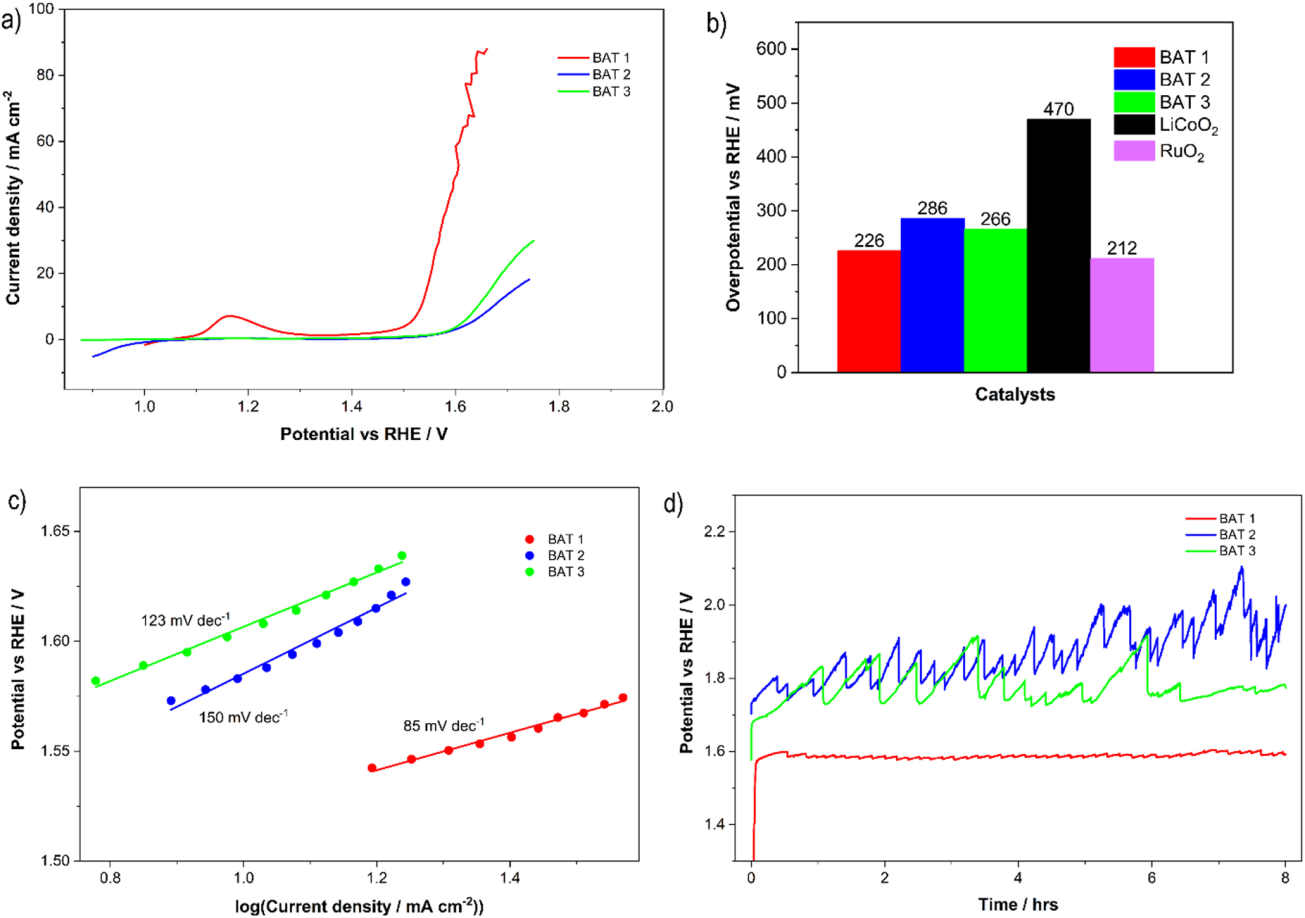
The electrochemical OER performance of battery waste BAT 1–3: (**a**) LSV curves after IR correction using EIS measurements; (**b**) overpotential at 10 mA cm^− 2^; (**c**) Tafel plots; (**d**) CP curves recorded in 0.1 M KOH at 10 mA cm^− 2^.

The electrocatalytic OER activity of GC electrodes modified with different post-leached battery waste was assessed using linear sweep voltammetry (LSV) and cyclic voltammetry (CV) techniques (Fig. 3). For comparison, the OER activity of benchmark LiCoO_2_ and RuO_2_ catalysts was also evaluated (Figures S3-S6 in SI). As shown in Fig. 3a, the LSV curves revealed that the battery waste material BAT 1 demonstrated superior OER activity compared to BAT 2, BAT 3, and the commercially available LiCoO₂ (Figure S6), which is commonly utilized in Li-ion battery manufacturing. The onset potential (*E*_onset_) required for reaching 10 mA cm^− 2^ was 1.46 V for BAT 1 (Fig. 3a) which is lower than either for BAT 2 (1.512 V), BAT 3 (1.5 V) or LiCoO_2_ (1.7 V) (Fig. 3a, b, Figure S6) and is only of 226 mV higher than the theoretical thermodynamic potential for water splitting (1.23 V vs. RHE). Moreover, OER onset potential for BAT 1 is just 14 mV higher than the benchmark RuO_2_ (Fig. 3b, Figure S4) but lower than for IrO_2_^57^ (Fig. 3a, b), and for Co_3_O_4_ (440 mV)^57^. Furthermore, BAT 1 demonstrated superior OER catalytic performance, with an overpotential of 226 mV to reach 10 mA cm^− 2^, outperforming graphite-based materials recycled from spent Li-ion batteries (506 mV and 436 mV)^58^. These materials had undergone additional chemical oxidation and N-doping post-recycling, which enhanced their catalytic properties^59^.

A comparison of the electrocatalytic properties of the battery waste materials under investigation reveals that BAT 1 exhibits the highest OER performance, while BAT 2 shows the lowest catalytic activity, with OER overpotentials of 226 mV and 286 mV at 10 mA cm^− 2^, respectively (Fig. 3b). Additionally, BAT 1 displays the lowest Tafel slope (85 mV dec^− 1^) among the battery waste materials studied (Fig. 3c), which is lower than that of RuO_2_ (Figure S4), suggesting a faster reaction rate. In contrast, LiCoO_2_, identified as a benchmark material, exhibits a relatively high overpotential at 10 mA cm^− 2^ and a higher Tafel slope (Figure S6), indicating the slowest OER reaction rate.

Additionally, the noticeable anodic peak that appears at about 1.18 V on the CV curve for BAT 1 indicates the presence of a significant number of redox-active cobalt compounds (Co^2+^/Co^3+^) at its surface as compared to other tested battery waste, which probably contain fewer bulk cobalt compounds. This conclusion aligns with the findings of Raman and XRD investigations, which show a predominance of Co-based compounds in BAT 1 compared to the other battery waste materials.

Furthermore, BAT 1 demonstrated robust durability, with no significant increase in electrode potential observed during long-term stability measurements conducted in 0.1 M KOH (Fig. 3d). The initial potential rise observed for the BAT 1 sample is more pronounced compared to the other catalysts. This behaviour is attributed not only to the temporary blockage of active sites by trapped oxygen bubbles, as commonly observed during oxygen evolution, but also to the porous nature of BAT 1. The increased surface area and pore volume may lead to double-layer charging effects and gradual wetting by the electrolyte. Additionally, the initial rise may reflect surface restructuring or activation processes that occur under applied current. After this brief initial phase, the potential stabilizes, indicating the formation of a steady and catalytically active interface. The long-term experiments performed for BAT 2 and BAT 3 revealed their lower stability affected more by the oxygen bubbles attached to/detached from the electrode surface.

**Fig. 4:**
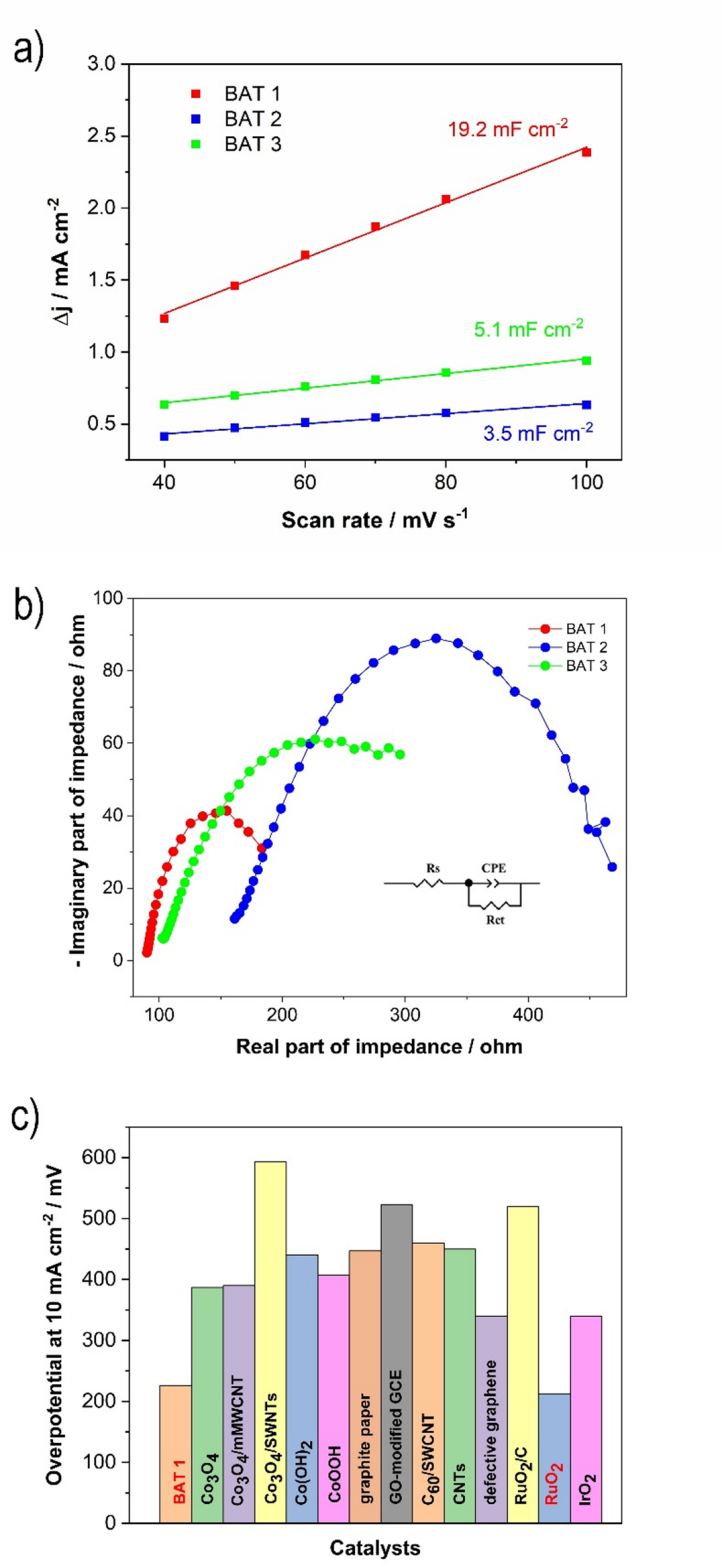
(**a**) Average current density (Δj=(j_a_-j_c_)/2) versus the scan rate, presenting the double-layer capacitance (C_dl_) taken from the corresponding CVs (Figure S8); (**b**) EIS spectra recorded at constant potential for reaching 10 mA cm^− 2^; (**c**) A comparison of the selected cobalt and carbon-based catalysts for OER in KOH electrolyte^61–64^..

The superior OER performance of BAT 1 compared to BAT 2 and BAT 3 (Figs. 3 and 4) can be attributed to clear differences in their chemical composition, oxidation states, and microstructure, which result from the distinct leaching conditions applied. BAT 1, leached in sulfuric acid, retained a higher number of cobalt-based phases, as evidenced by XRD and Raman analysis, which correlates with the prominent anodic peak observed at ca. 1.18 V in the CV curves. This peak indicates the presence of redox-active Co^2+^/Co^3+^ species known to enhance OER kinetics. Furthermore, BAT 1 exhibits a more porous morphology than BAT 2 and BAT 3, which is supported by electrochemically active surface area (ECSA) analysis (ECSA = C_dl_/C_s_, where C_dl_ is a double layer capacitance, and C_s_ - a specific capacitance of the electrode)^36^ and as we compared similarly compositional battery waste materials (based on carbon black mass with slightly different metal contents), the ECSA values for battery waste materials changed in the following manner: BAT 1 > BAT 3 > BAT 2 (Fig. 4a). The increased porosity facilitates better diffusion of electrolyte and more effective exposure of catalytic sites. These factors, higher Co content, accessible redox pairs, and improved microstructure, synergistically enhance the electrocatalytic activity of BAT 1, distinguishing it from the other battery-derived catalysts.

To examine the OER kinetics, the EIS and the corresponding simulated electrical equivalent circuit model (Fig. 4b) were employed. The charge transfer resistance of BAT 1 is 110.7 ohms, which is much lower than that of other battery waste (342.2 ohms and 314.5 ohms for BAT 2 and BAT 3, respectively), suggesting that this material has faster charge transfer processes (Table S1 in SI).

Figure 4 illustrates that BAT 1 exhibits superior OER electrocatalytic activity to that of most of the recently studied water OER catalysts, including cobalt oxides and cobalt carbon-based materials, and only slightly lower than for RuO_2_.

### Seawater splitting

**Fig. 5:**
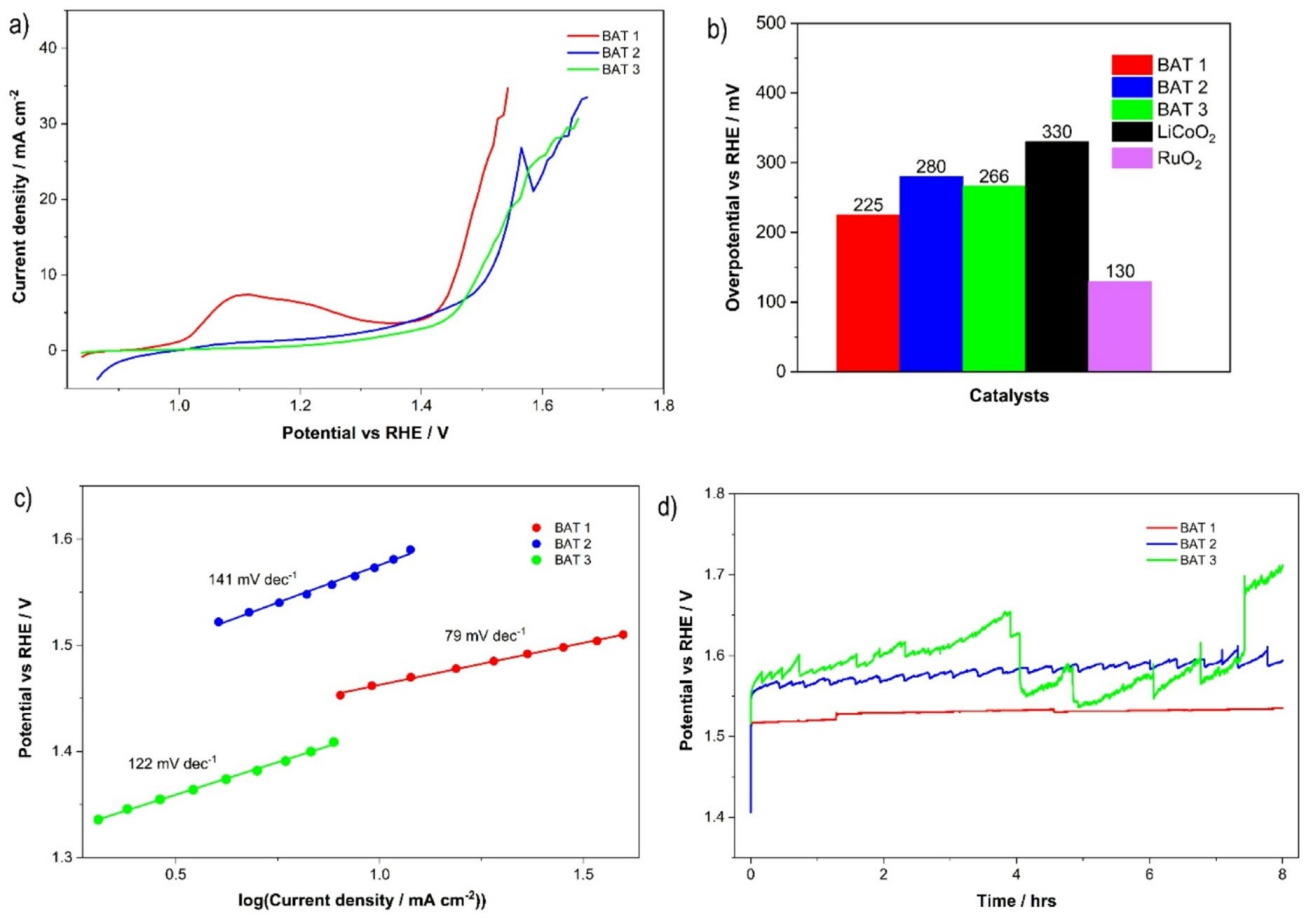
The electrochemical OER performance of battery waste BAT 1–3: (**a**) LSV curves after IR correction using EIS measurements; (**b**) overpotential at 10 mA cm^− 2^; (**c**) Tafel plots; (**d**) CP curves recorded in 1 M KOH + 1 M NaCl (1:1 vol.) at 10 mA cm.

Following the confirmation of the robust OER electrocatalytic activity of Li-ion battery waste in alkaline freshwater electrolyte, its OER performance was further explored in an alkaline simulated seawater electrolyte (1 M KOH : 1 M NaCl, 1:1 vol.). As shown in Fig. 5, the OER performance of different battery waste materials (BAT 1*–*3) exhibited slight variations. BAT 2 demonstrated the lowest OER performance, consistent with findings from freshwater splitting studies. It exhibited an overpotential of 280 mV at 10 mA cm^− 2^, along with a higher Tafel slope, indicating slower OER kinetics compared to the other battery waste materials. In contrast, BAT 1 showed the lowest overpotential (225 mV) to achieve 10 mA cm^− 2^, while BAT 3 required 266 mV of overpotential for the same current density. BAT 1 exhibited a lower Tafel slope (79 mV dec^− 1^) compared to BAT 2 and BAT 3 (141 mV dec^− 1^ and 122 mV dec^− 1^, respectively), suggesting that the OER is more facile with BAT 1. Two benchmark materials, RuO_2_ and LiCoO_2_, demonstrated lower and higher overpotentials for the OER, respectively, with values of 130 mV and 330 mV, respectively (see Figure S5 and Figure S7). However, the relatively poor OER kinetics of LiCoO_2_ and RuO_2_ in seawater splitting is evidenced by higher Tafel slope values (101 mV dec^− 1^ and 182 mV dec^− 1^, respectively) than for BAT 1. In general, the Tafel slopes determined for tested battery waste Materials as well as the benchmark catalysts differ than for those obtained in 0.1 M KOH electrolyte, indicating different rate-determining steps within a given pathway^64,65^. The OH^−^ electrosorption on the electrocatalyst surface initiates a typical oxygen generation in the water/seawater splitting process. Therefore, a high affinity for adsorbed OH-intermediates is a necessary feature of an effective catalyst with high OER performance. Then, the subsequent steps of oxygen generation will become rate-determining steps if the formation and equilibrium coverage of OH-intermediates are rapidly reached, leading to a smaller Tafel slope^65^.

Additionally, the LSV curves for BAT 1 (at 1.1 V, Fig. 5a) clearly show an anodic peak corresponding to the redox Co²⁺/Co³⁺ couple, similar to that observed in 0.1 M KOH electrolyte. This anodic peak is more visible than that observed in 0.1 M KOH, which may result from the chloride ions present in the alkaline electrolyte that enhance the cobalt oxidation reaction^66^.

The chronopotentiometry experiments conducted at a current density of 10 mA cm^− 2^ (Fig. 5d) did not reveal any discernible decrease in BAT 1 activity following 8 h of stability tests. Only a few notable electrode potential increases/decays are related to the observed formation of oxygen bubbles, which attach to the electrode surface. BAT 2 and BAT 3 exhibited lower stability manifested by the variation of the electrode potentials due to the oxygen bubble formation, similarly to the experiments performed in the alkaline solutions.

**Fig. 6:**
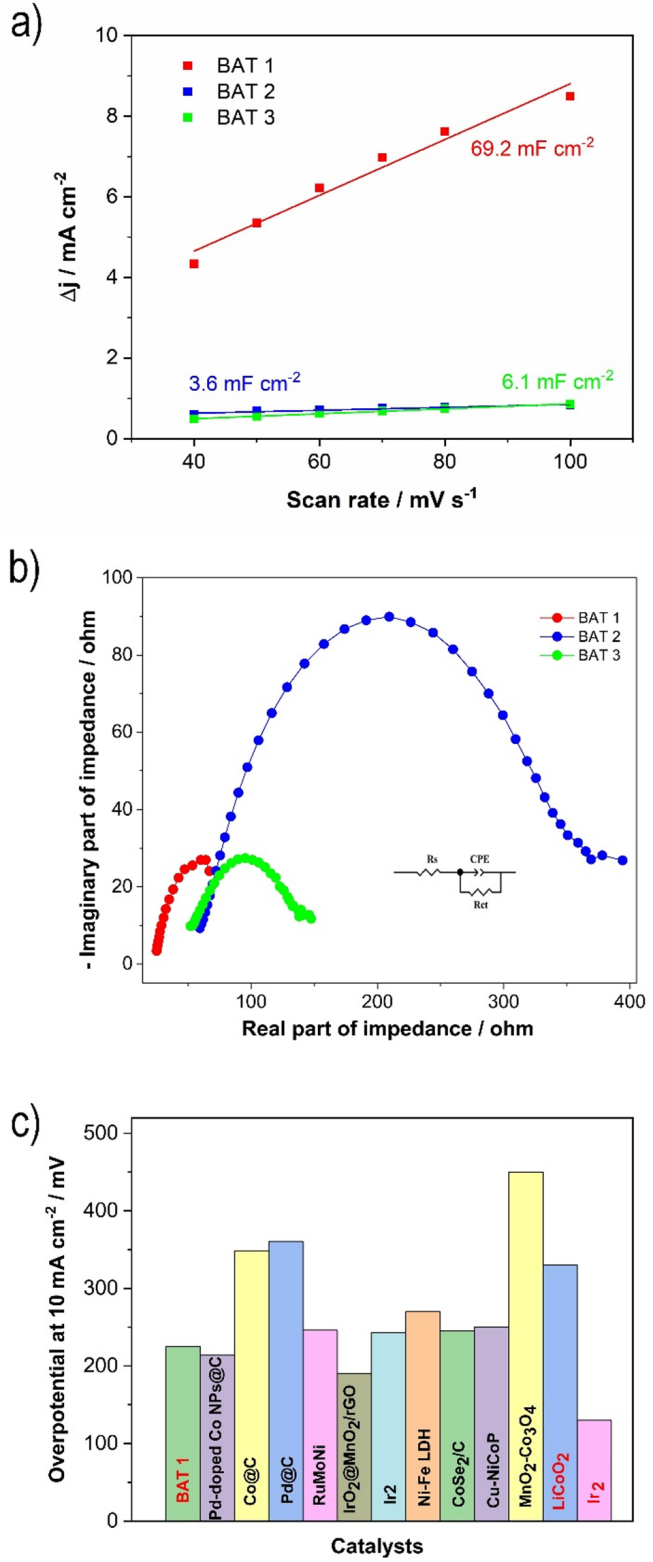
(**a**) Average current density (Δj=(ja-jc)/2) versus the scan rate, presenting the double-layer capacitance (Cdl) taken from the corresponding CVs (Figure S8); (**b**) EIS spectra recorded at constant potential for reaching 10 mA cm^− 2^; (**c**) A comparison of OER overpotential for reaching of 10 mA cm^− 2^of various catalysts in KOH + NaCl (or KOH + seawater) solution^68–73]^.

Figure 6a shows the double layer capacitance C_dl_ determined for battery waste materials tested in the simulated seawater medium according to the same procedure as previously utilized for OER in KOH electrolyte. As can be seen, the same trend is observed: BAT 1 exhibits the highest C_dl_ (69.2 mF cm^− 2^), while BAT 2 exhibits the lowest (3.6 mF cm^− 2^). That means the ESCA for the tested materials changes as follows: BAT 1 > BAT 3 > BAT 2.

The EIS and the corresponding simulated electrical equivalent circuit model (Fig. 6b) employed to study the OER kinetics showed that BAT 1 has a lower charge transfer resistance (67.4 ohm) than BAT 2 (322 ohm) and BAT 3 (111 ohm).

As depicted in Fig. 6c, the OER performance of BAT 1 is superior to that of the majority of recently explored seawater OER catalysts, including carbon-transition metal-based composites. However, it exhibits slightly inferior OER characteristics in comparison to noble metal oxides and composites, demonstrating an overpotential of 95 mV higher than that observed for RuO_2_.

## Conclusions

This study represents a significant advancement in our understanding of the catalytic potential of post-leached battery waste powders, specifically the residual black carbon mass that remains after metal recovery. Our findings demonstrate that these powders can efficiently drive the oxygen evolution reaction (OER) for seawater splitting with remarkably low overpotential. The structure, morphology, and composition of the spent BAT 1–3 powders were found to be markedly influenced by the leaching conditions. It is noteworthy that the leaching process using sulfuric acid (BAT 1) resulted in the lowest recovery rate of cobalt, yet the highest efficiency in the catalytic process for the OER, both in water and seawater splitting. This highlights the crucial role of cobalt-based materials in this reaction. Moreover, the recovery process undergone with sulfuric acid has resulted in a more developed battery waste structure exhibiting a higher electrochemical surface active area (BAT 1) than obtained after recovery with organic acids (BAT 2 and BAT 3). These findings indicate that a well-developed surface structure is a key factor in enhancing the efficiency of electrocatalytic water splitting in saline environments.

Detailed characterization confirmed that BAT 1 is composed of higher content of cobalt-based compounds, such as LiCoO_2_ and Co_3_O_4_, uniformly dispersed within a porous and conductive carbon matrix. This unique combination provided a higher electrochemically active surface area compared with powders obtained from organic acid leaching (BAT 2 and BAT 3). These structural and compositional features explain the superior electrocatalytic activity of BAT 1.

The results of electrochemical tests, coupled with compositional and structural analyses, revealed that the presence of LiCoO₂ and other cobalt-based compounds, as well as a highly porous surface structure (electrochemically active surface area), are critical factors influencing the OER catalytic performance of battery waste. Conversely, higher levels of crystallinity were found to contribute less significantly to catalytic activity. It is noteworthy that the post-leached battery waste powders exhibited remarkable OER electrocatalytic activity in water and seawater splitting, exceeding that of a widely studied carbon–transition metal-based material. Electrochemical tests further revealed that BAT 1 reached an OER overpotential only 14 mV and 95 mV higher than benchmark RuO₂ catalyst for water and seawater splitting, respectively. These findings highlight the potential of waste-derived, low-cost electrocatalysts in advancing the hydrogen economy.

In summary, the electrocatalytic performance of spent lithium-ion batteries (LiBs) is determined not only by the cobalt-based compound content but also by the morphology of the carbon-based matrix. It is demonstrated by BAT 1 that the optimal balance of both of these factors is achieved. This study provides valuable insights into the role of these factors in energy conversion processes and represents a significant step toward the reuse of spent LiBs. The findings emphasize the critical importance of recycling battery waste powders for electrocatalytic applications, contributing to a circular economy and the sustainable utilization of resources.

## Supplementary Information

Below is the link to the electronic supplementary material.

Supplementary Material 1

## Data Availability

The data presented in this study are available in ZENODO at 10.5281/zenodo.17357380, accessed on 15 October 2025, and in RepOD at10.18150/0BULBV, accessed on 08 October 2025.
