# Supplementary material for: Lithium-ion battery waste as a robust oxygen evolution reaction electrocatalyst for seawater splitting
Source: Sci Rep. 2026 Jan 12;16:4688. doi: 10.1038/s41598-025-34856-w (PMC12868884; doi:10.1038/s41598-025-34856-w)
Supplement: Supplementary file 1 — Supplementary Material 1 [file 41598_2025_34856_MOESM1_ESM.docx]

SUPPLEMENTARY

Lithium-Ion Battery Waste as a Robust Oxygen Evolution Reaction Electrocatalyst for Seawater Splitting

*Magdalena Warczak ^1*^, Katarzyna Belka ^1^, Weronika Urbańska ^2^, Monika Michalska ^3^, Njemuwa Nwaji ^4^, Magdalena Osial ^4^*

^1^ Bydgoszcz University of Science and Technology, Faculty of Chemical Technology and Engineering, Seminaryjna 3 Street,85-326 Bydgoszcz, Poland; [magdalena.warczak@pbs.edu.pl](mailto:magdalena.warczak@pbs.edu.pl)

^2^ Wrocław University of Science and Technology, Faculty of Environmental Engineering, Wybrzeże Wyspiańskiego 27 Street, 50-370 Wrocław, Poland

^3^ VSB-Technical University of Ostrava, Faculty of Materials Science and Technology, Department of Chemistry and Physico-Chemical Processes, 17. listopadu 2172/15, 708 00, Ostrava-Poruba, Czech Republic

^4^ Institute of Fundamental Technological Research, Polish Academy of Sciences, Department of the Theory of Continuous Media and Nanostructures, Pawińskiego 5B Street, 02-106 Warsaw, Poland

E-mail: [magdalena.warczak@pbs.edu.pl](mailto:magdalena.warczak@pbs.edu.pl) (corresponding author)


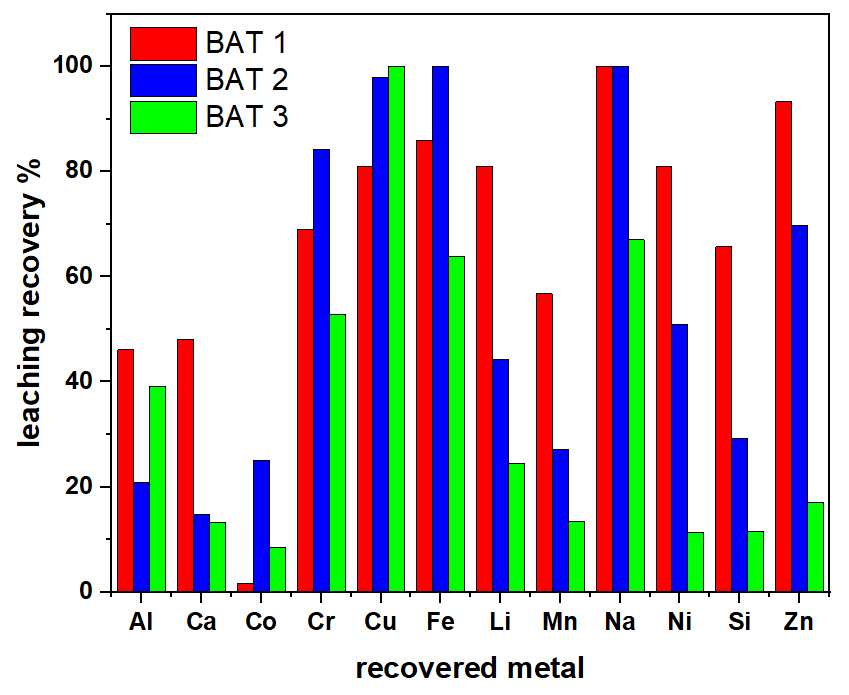


**Figure S1. ICP-MS recovery rate of metals for BAT 1-3.**

**
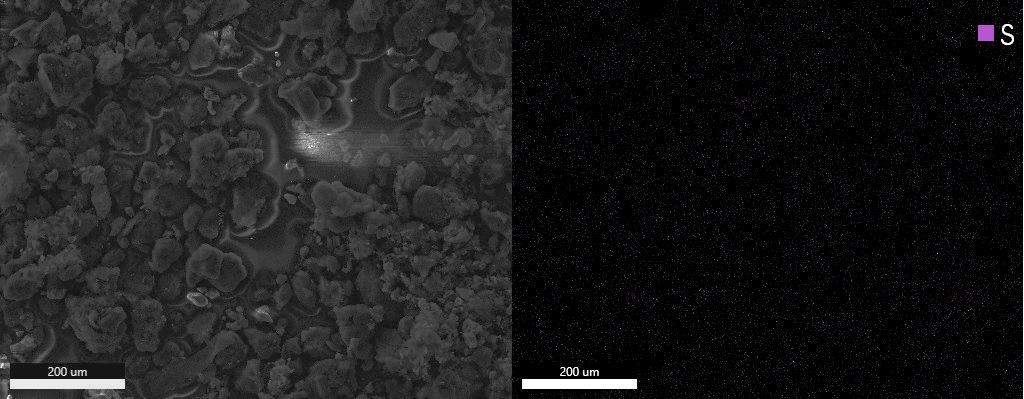
**

**Figure S2. SEM image and sulphur EDS map of BAT 1.**

**
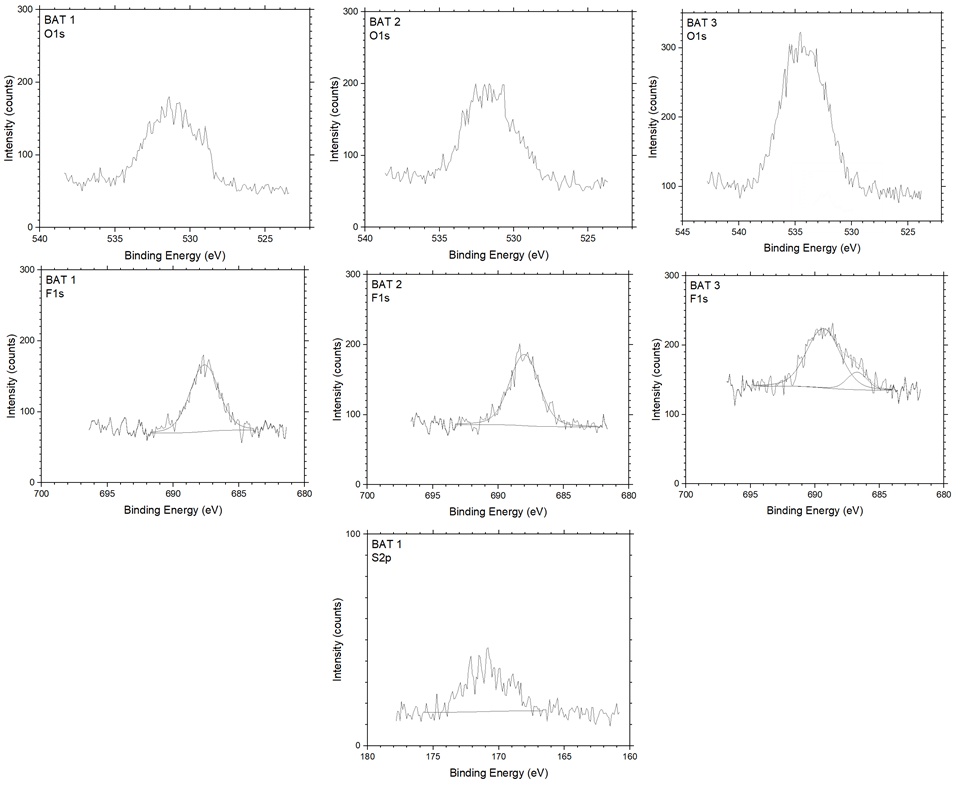
**

**Figure S3. High-resolution O 1s, F 1s XPS spectra for BAT 1-3 and S 2p spectrum for BAT 1.**

**
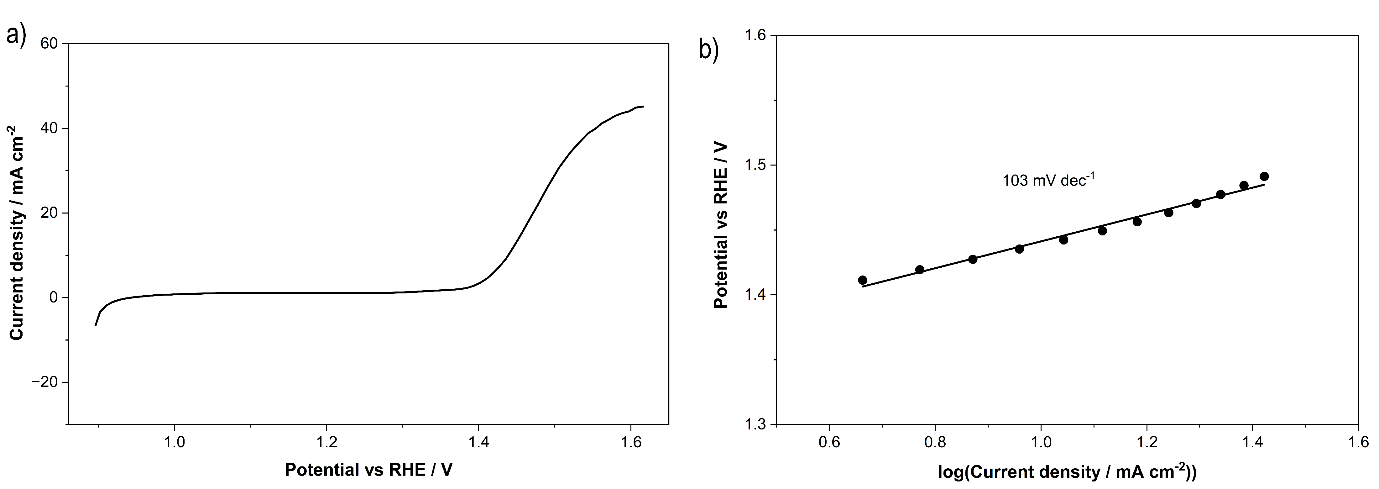
**

**Figure S4. The electrochemical OER performance of benchmark RuO_2_ (a) LSV curve after IR correction using EIS measurements, and (b) Tafel plot recorded in 0.1 M KOH.**

**
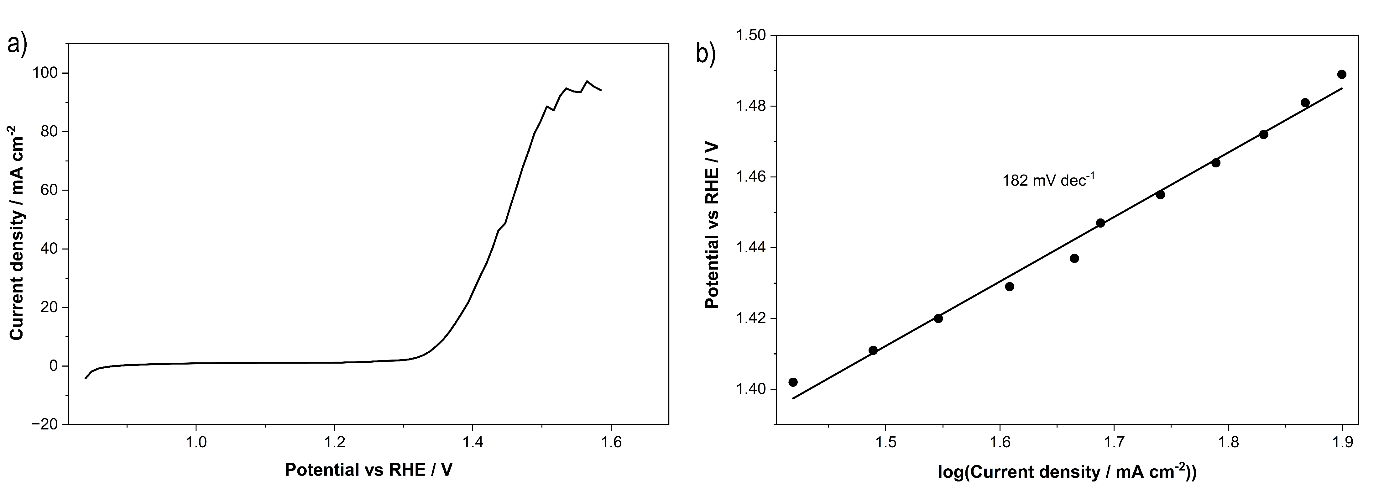
**

**Figure S5. The electrochemical OER performance of benchmark RuO_2_ (a) LSV curve after IR correction using EIS measurements, and (b) Tafel plot recorded in 1 M KOH + 1 M NaCl (1:1 vol.).**

**
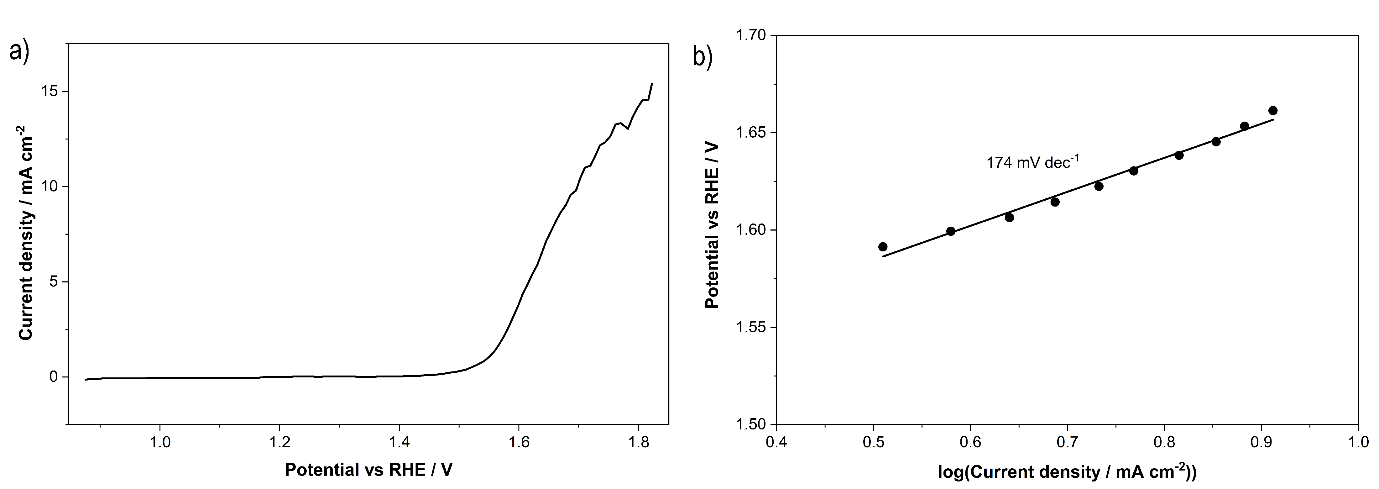
**

**Figure S6. The electrochemical OER performance of benchmark LiCoO_2_ (a) LSV curve after IR correction using EIS measurements, and (b) Tafel plot recorded in 0.1 M KOH.**


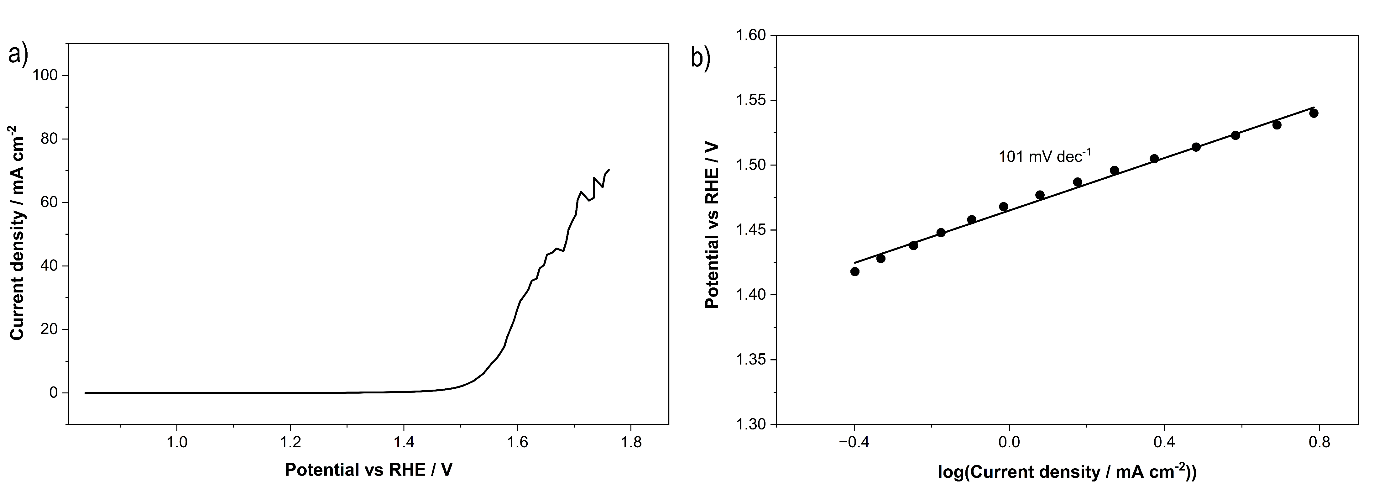


**Figure S7. The electrochemical OER performance of benchmark LiCoO_2_ (a) LSV curve after IR correction using EIS measurements, and (b) Tafel plot recorded in 1 M KOH + 1 M NaCl (1:1 vol.).**


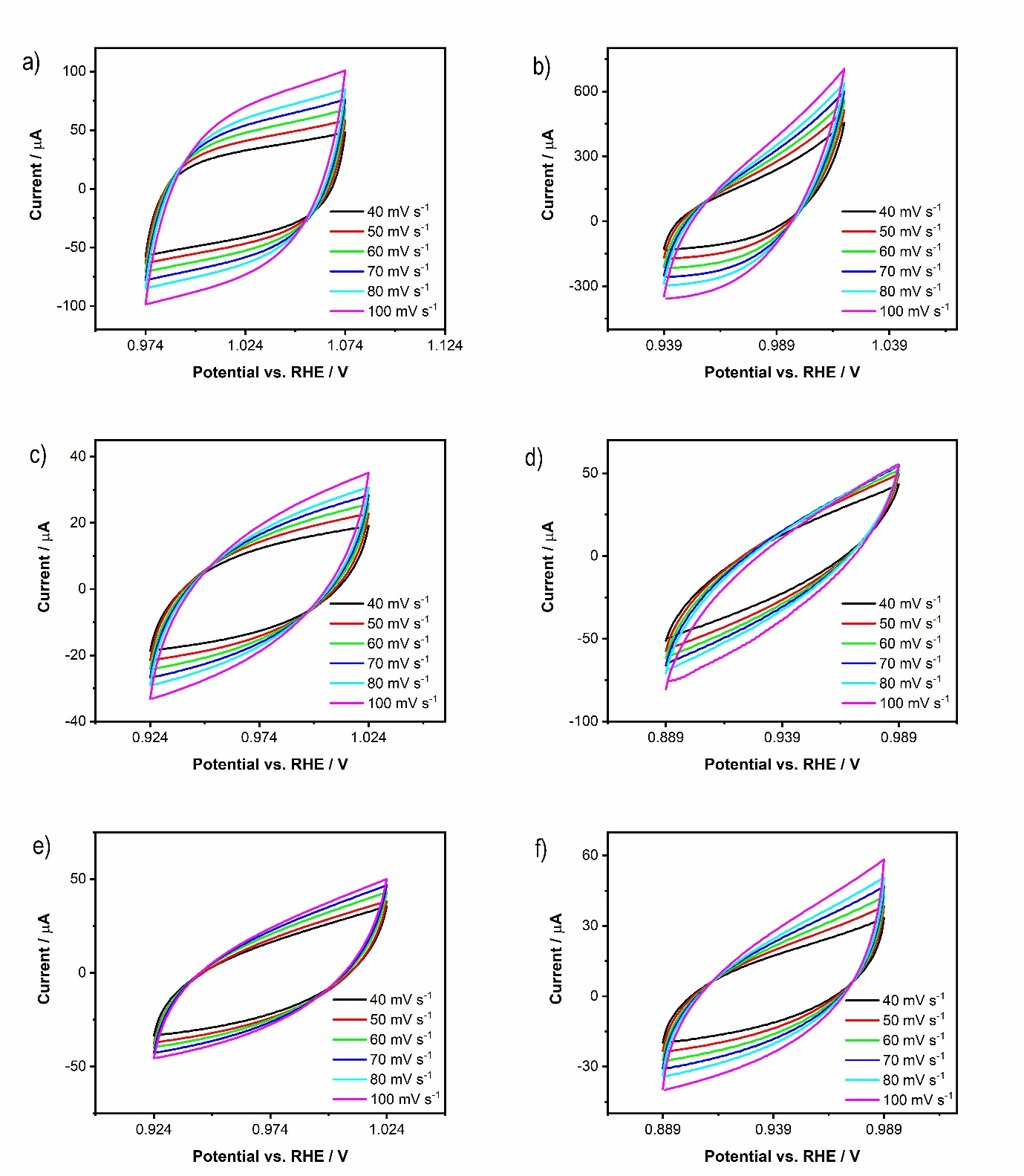


**Figure S8. Cyclic voltammograms of GC electrodes modified by various battery waste: BAT 1 (a,b), BAT 2 (c,d) and BAT 3 (e,f) recorded in 0.1 M KOH (left columns) and in 1 M KOH + 1 M NaCl (1:1 vol) (right columns) at various scan rates.**

**Table S1. Fitted parameters of equivalent circuit model obtained for EIS data recorded in 0.1 M KOH and 1M KOH + 1 M NaCl: R_s_ – electrolyte resistance, CPE – constant phase element referring to double layer capacitance, and R_ct_ – charge transfer resistance.**

|  | **0.1 M KOH** | | | **1 M KOH + 1 M NaCl** | | |
| --- | --- | --- | --- | --- | --- | --- |
| **Element** | **BAT 1** | **BAT 2** | **BAT 3** | **BAT 1** | **BAT 2** | **BAT 3** |
| Rs | 89.34 | 156.1 | 96.05 | 24.53 | 54.38 | 43.38 |
| CPE-T | 0.0318 | 0.0012 | 0.0038 | 0.0797 | 0.0010 | 0.0018 |
| CPE-P | 0.83 | 0.61 | 0.5 | 0.84 | 0.66 | 0.56 |
| Rct | 110.7 | 342.2 | 314.5 | 67.64 | 322 | 111 |

 ** **     
